# Antigen-Specific Interferon-Gamma Responses and Innate Cytokine Balance in TB-IRIS

**DOI:** 10.1371/journal.pone.0113101

**Published:** 2014-11-21

**Authors:** Odin Goovaerts, Wim Jennes, Marguerite Massinga-Loembé, Ann Ceulemans, William Worodria, Harriet Mayanja-Kizza, Robert Colebunders, Luc Kestens, for the TB-IRIS Study Group

**Affiliations:** 1 Department of Biomedical Sciences, Institute of Tropical Medicine, Antwerp, Belgium; 2 Department of Biomedical Sciences, University of Antwerp, Antwerp, Belgium; 3 Centre de Recherches Médicales de Lambaréné (CERMEL), Albert Schweitzer Hospital, Lambaréné, Gabon; 4 Institut für Tropenmedizin, Universität Tübingen, Tübingen, Baden-Württemberg, Germany; 5 Makerere University College of Health Sciences, Kampala, Uganda; 6 Infectious Diseases Institute, Makerere University College of Health Sciences, Kampala, Uganda; 7 Infectious Diseases Network for Treatment and Research in Africa (INTERACT), Kampala, Uganda; 8 Department of Clinical Sciences, Institute of Tropical Medicine, Antwerp, Belgium; 9 Epidemiology and Social Medicine, University of Antwerp, Antwerp, Belgium; National Institute of Infectious Diseases, Japan

## Abstract

***Background*:**

Tuberculosis-associated immune reconstitution inflammatory syndrome (TB-IRIS) remains a poorly understood complication in HIV-TB patients receiving antiretroviral therapy (ART). TB-IRIS could be associated with an exaggerated immune response to TB-antigens. We compared the recovery of IFNγ responses to recall and TB-antigens and explored in vitro innate cytokine production in TB-IRIS patients.

***Methods*:**

In a prospective cohort study of HIV-TB co-infected patients treated for TB before ART initiation, we compared 18 patients who developed TB-IRIS with 18 non-IRIS controls matched for age, sex and CD4 count. We analyzed IFNγ ELISpot responses to CMV, influenza, TB and LPS before ART and during TB-IRIS. CMV and LPS stimulated ELISpot supernatants were subsequently evaluated for production of IL-12p70, IL-6, TNFα and IL-10 by Luminex.

***Results*:**

Before ART, all responses were similar between TB-IRIS patients and non-IRIS controls. During TB-IRIS, IFNγ responses to TB and influenza antigens were comparable between TB-IRIS patients and non-IRIS controls, but responses to CMV and LPS remained significantly lower in TB-IRIS patients. Production of innate cytokines was similar between TB-IRIS patients and non-IRIS controls. However, upon LPS stimulation, IL-6/IL-10 and TNFα/IL-10 ratios were increased in TB-IRIS patients compared to non-IRIS controls.

***Conclusion*:**

TB-IRIS patients did not display excessive IFNγ responses to TB-antigens. In contrast, the reconstitution of CMV and LPS responses was delayed in the TB-IRIS group. For LPS, this was linked with a pro-inflammatory shift in the innate cytokine balance. These data are in support of a prominent role of the innate immune system in TB-IRIS.

## Introduction

Together with the HIV pandemic there has been a global increase in the number of tuberculosis (TB) infections [1]. An estimated 14 million individuals are dually infected with HIV and TB worldwide [2]. Despite recent WHO recommendations for early antiretroviral therapy (ART) [3], treatment is started at late stages of HIV infection in many developing countries [4]. This puts patients at increased risk of developing tuberculosis-associated immune reconstitution inflammatory syndrome (TB-IRIS) during ART [5]. TB-IRIS presents in up to 25% of HIV-TB patients as worsening symptoms of TB during ART, despite a favourable response to TB-treatment (hence the name “paradoxical TB-IRIS”) [6]. This complication typically occurs within the first 2 months after starting ART, with the majority occurring within the first few weeks [7]. TB-IRIS poses a significant diagnostic challenge to physicians and reliable laboratory markers to help detect this syndrome are urgently needed [8].

Known risk factors of TB-IRIS include a low CD4 count, high TB-antigen burden and short interval between initiation of TB treatment and ART [9], [10]. The pathogenesis of TB-IRIS remains largely unclear, although there are clear signs of tissue-destructive inflammation during immune reconstitution (reviewed in [11], [12]). This process could involve an amplified immune response to TB bacilli or their residual antigens [11], [13]. Early research suggested that TB-IRIS development was linked to elevated T-helper type 1 (Th1) responses to TB-antigens. Indeed, studies in TB-IRIS patients reported elevated IFNγ responses to several TB-associated antigenic compounds such as purified protein derivative (PPD), 6 kDa early secretory antigenic target (ESAT-6) and 10 kDa culture filtrate antigen (CFP-10) [14]–[18]. However, elevated IFNγ responses to TB-antigens are often also seen in HIV-TB patients who do not develop TB-IRIS [19]–[21], casting doubt on the causal role of Th1 cells in TB-IRIS pathogenesis. It has been suggested that disturbances in the innate immune system [22] or in the interplay between the innate and adaptive immune system [11] could drive TB-IRIS pathogenesis. This is supported by repeated findings of elevated levels of IL-6 and TNFα, among other innate cytokines, during TB-IRIS [14], [16], [23]–[26]. Interestingly, elevated innate cytokine production has also been reported in TB-IRIS patients after toll-like receptor (TLR) 2 stimulation with lipomannan, a TB pathogen-associated molecular pattern (PAMP) [27]. Put together, there is evidence that responses to antigenic stimulation are unbalanced in TB-IRIS, both in the innate and the adaptive arm of the immune system. However, the roles of antigen-specific IFNγ production and TLR stimulation in TB-IRIS remain to be completely elucidated.

In this study, we aimed to assess the ART related recovery of IFNγ responses in TB-IRIS patients. To this end, we determined specific responses to a number of TB and recall antigens in a well matched selection of TB-IRIS patients and controls from a large prospective cohort [28]. In addition, we explored the possible innate component of TB-IRIS by studying cytokine production upon TLR4 stimulation with lipopolysaccharide (LPS). We report a disturbed reconstitution of the IFNγ recall response, without an excessive IFNγ response to TB-antigens. In addition, we observed a pro-inflammatory shift in the innate cytokine balance upon LPS stimulation during TB-IRIS, providing evidence of the involvement of the innate immune system in TB-IRIS.

## Materials and Methods

### Study population

Patients were recruited in a prospective observational study on paradoxical TB-IRIS at Mulago Hospital, Kampala, Uganda, between January 2008 and July 2010 as described previously [9], [28], [29]. The present study is based on HIV-TB co-infected adults who were being treated for active TB infection and put on ART within 2 months after starting TB treatment (median 6 weeks prior to ART). All HIV-patients received non-nucleoside reverse transcriptase inhibitor-based ART according to Ugandan national guidelines and were monitored for paradoxical TB-IRIS development during at least 3 months. Blood samples were taken before ART initiation (pre-ART) and when patients were diagnosed for TB-IRIS (IRIS event). Patients without IRIS-related symptoms were used as non-IRIS controls and had samples taken pre-ART, at 2 weeks and 1 month on ART. To compare with the expected immunocompetent antigen responses, two additional groups of HIV-uninfected subjects were recruited. One group was receiving treatment for active TB for less than 4 months (HIV−TB+ controls) while the other had no clinical signs of active TB (HIV−TB− controls). HIV-uninfected subjects had samples taken only once.

### Patient selection and matching

A large majority of patients from our cohort developed TB-IRIS within 1 month after ART initiation. To limit heterogeneity among TB-IRIS patients, we included only patients who developed TB-IRIS within one month on ART. This stricter selection of patients reduces potential bias due to differences in kinetics and immunopathology between early- and late-onset TB-IRIS. Selected TB-IRIS patients with PBMCs available pre-ART and at occurrence of IRIS were matched 1 by 1 with non-IRIS controls for sex, baseline CD4 count (+/− 15 CD4 cells/mm^3^) and age (≤10 years difference). Samples from non-IRIS controls, serving as a control time point for their paired IRIS event, were selected at either 2 weeks or 1 month on ART. This selective pairing allowed for a matched time on ART between TB-IRIS patients and non-IRIS controls.

### Definitions

*Mycobacterium tuberculosis* infection was diagnosed according to the TB/HIV WHO guidelines [30]. The diagnostic evaluation for TB included: clinical examination, chest X-rays and abdominal ultrasounds, sputum smear microscopy for acid-fast bacilli and mycobacterial culture of sputum, aspirate or effusion if available. TB-IRIS cases were classified by a committee of two co-authors (RC and WW) after reviewing all suspected TB-IRIS cases evaluated according to the International Network for the Study of HIV-associated IRIS (INSHI) clinical case-definition for resource limited settings [5]. This evaluation included: a symptom questionnaire, detailed physical examination to confirm TB-IRIS and exclude alternative causes and comparison of a second chest X-ray and abdominal ultrasound scan to the patient's baseline examination. Biological TB-IRIS samples collected when patients developed new or worsening symptoms following initiation of ART. These symptoms included at least 1 major criterion, such as enlarged lymph nodes, or 2 minor criteria, such as fever and cough.

### ELISpot assays

PBMCs were collected from TB-IRIS patients and cryopreserved in liquid nitrogen. Samples were consequently thawed and IFNγ responses to antigens were measured by ELISpot (Diaclone SAS, Besançon Cedex, France) according to the manufacturer's instructions. In brief, PBMCs were cultured overnight in an antibody-coated ELISpot filter plate at 200,000 cells/200 µl in RMPI+2.5% human serum in the presence of either 10 µg/ml cytomegalovirus (CMV) lysate (Institut Virion\Serion GmbH, Würzburg, Germany), 5 µg/ml whole influenza virus antigen (H3N2 A/Sydney/5/97, National Institute for Biological Standards and Control, Hertfordshire, Great Britain), 10 µg/ml PPD (Statens Serum Institute, Copenhagen, Denmark), 10 µg/ml recombinant ESAT-6 (Statens Serum Institute, Copenhagen, Denmark), and 10 µg/ml recombinant CFP-10 (a kind gift from Lionex Diagnostics and Therapeutics, Braunschweig, Germany). In addition, 100 ng/ml LPS (*E. coli* O55:B5, Sigma-Aldrich BVBA, Diegem, Belgium) was included separately as a strong inducer of innate cytokines. Medium only and 5 µg/ml staphylococcal enterotoxin B (SEB, Sigma-Aldrich BVBA, Diegem, Belgium) were used as negative and positive controls, respectively. The number of IFNγ spot-forming cells (SFC) per 10^6^ PBMCs was determined using the ELISpot Reader and ELISpot Reader software (AID, Strassberg, Germany). Data from unstimulated medium controls was subtracted before reporting the number of SFC. Because we directly compared quantitative responses between patient groups, no criteria were used for defining positive responses.

### Cytokine multiplex assay

ELISpot supernatants from PBMCs stimulated with CMV and LPS were stored at −80°C until further use. Samples were thawed within 1 hour prior to analysing cytokine levels by using the Bio-Plex human cytokine assay kits (Bio-Rad Laboratories NV-SA, Nazareth, Belgium) according to the manufacturer's instructions. We measured in vitro levels of IL-6, TNFα and IL-10 as a representation of innate pro- and anti-inflammatory cytokine production. In addition, IL-12p70 was measured as a link between changes in IFNγ responses and monocyte function. Samples were diluted 2× for TNFα and IL-10 and 10× for IL-6 to allow optimal detection of both high and low cytokine concentrations.

### Ethical considerations

The study was approved by the Research Committee of the Infectious Diseases Institute (IDI), the ethical review board of Makerere University School of Medicine, the Uganda National Council of Science and Technology and by the institutional review board of the Institute of Tropical Medicine of Antwerp and the Ethics Committees of the Faculties of Medicine of the University of Antwerp. Written informed consent was obtained from all study participants.

### Statistical analysis

Differences between paired patients and changes in concentration over time for each patient were analysed using the Wilcoxon signed-rank test for paired data. Differences between HIV-infected and HIV-uninfected patients were analysed using the Mann-Whitney U test. Correlations were calculated using the Spearman's rank correlation. Statistics were performed using SPSS software (version 17.0) or GraphPad Prism (version 5) with significance level set at p<0.05. Because of the hypothesis driven nature of this study, no correction for multiple testing was applied [31], [32].

## Results

### Study population

A total of 18 TB-IRIS patients were paired with 18 non-IRIS controls (Table 1). TB-IRIS patients and non-IRIS controls did not differ regarding baseline viral load, TB treatment duration prior to ART, or ART duration prior to IRIS event or corresponding control time point. Twenty two HIV uninfected controls were included in the study, of whom 9 were being treated for TB (HIV-TB+ controls) and 13 did not have symptoms of TB (HIV-TB- controls). Neither HIV-TB+ nor HIV-TB- controls differed from TB-IRIS patients or non-IRIS controls regarding age or sex.

**Table 1:** Characteristics of TB-IRIS patients and matched controls.

Characteristics	TB IRIS (n = 18)	non-IRIS controls (n = 18)	p^a^
***Prior to ART***			
Male sex, n (%)	8 (44)	8 (44)	0.815^b^
Age (years)	34 (33–43)	40 (31–44)	0.760
CD4 (cell/mm^3^)	19 (11–119)	23 (8–93)	0.477
TB treatment duration prior to ART (days)	36 (23–56)	46 (23–58)	0.663
Viral load (log copies/ml)^c^	5.4 (5.3–5.8)	5.53 (5.1–5.6)	0.646
***During ART***			
Days between start of ART and TB-IRIS/control event	14 (12–22)	16 (14–28)	0.297

Values are shown as median values with interquartile range. The level of significance was set to p<0.05 for all tests. TB-IRIS patients were matched to non-IRIS controls for baseline CD4 count, age and sex.

*^a^* Wilcoxon signed-rank test.

*^b^* Mc Nemar test for binominal data.

*^c^* n = 10.

### Antigen-specific IFNγ ELISpot responses in HIV-TB patients developing TB-IRIS

**Figure 1:**
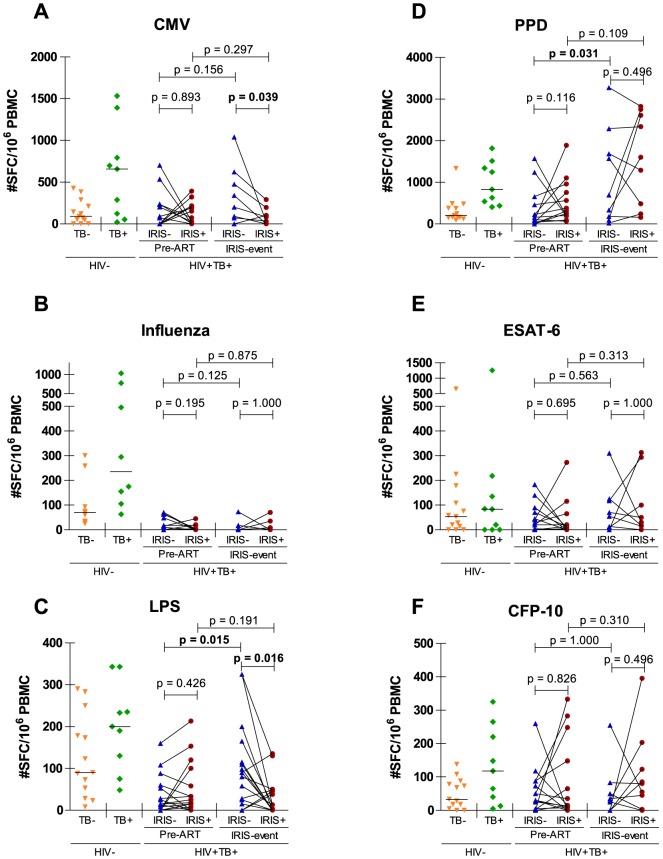
Antigen-specific IFNγ responses in TB-IRIS patients and controls. Dots on these graphs represent IFNγ spot-forming cells per 10^6^ PBMCs in TB-IRIS patients (IRIS+) and non-IRIS controls (IRIS-) after stimulation with CMV lysate (A), influenza antigen A (B), LPS (C), PPD (D), ESAT-6 (E) and CFP-10 (F). Dots connected with full lines represent matched patient pairs. Horizontal full lines represent median values for HIV-TB+ controls and HIV-TB- controls. Horizontal capped lines represent statistical comparisons between matched patients or between time points. The level of significance was set to p<0.05. A Wilcoxon signed-rank test was used to calculate p values between matched HIV patients and time points. Due to limited availability of viable PBMCs and pairwise exclusion, the number of patients across antigens and time points differed. Number of patients pre-ART were; 13 (A), 10 (B), 16 (C), 14 (D), 14 (E) and 14 (F). Number of patients during IRIS event were; 8 (A), 6 (B), 16 (C), 9 (D), 9 (E) and 9 (F).

We examined IFNγ responses to TB-antigens, CMV, Influenza and LPS (a well-known TLR4 antagonist) in order to assess the antigen-specific and non-specific immunity in TB-IRIS patients compared to non-IRIS, HIV-TB+ and HIV-TB- controls (Figure 1). At pre-ART, we observed no difference in IFNγ responses between TB-IRIS patients and non-IRIS controls for any of the antigens tested. Both groups showed a diminished IFNγ response to PPD, CMV, Influenza and LPS when compared to HIV-TB+ controls (p≤0.027) and to LPS and influenza when compared to HIV-TB- controls (p≤0.015).

IFNγ responses to the different TB-antigens were also similar between TB-IRIS patients and non-IRIS controls at IRIS event (Figure 1). Both groups showed signs of higher PPD responses compared to before ART. This increase was statistically significant for non-IRIS controls (p = 0.031), while TB-IRIS patients showed a trend (p = 0.109). Both groups also showed signs of higher PPD responses compared HIV-TB- controls, yielding significant results for TB-IRIS patients only (p = 0.007). In contrast, IFNγ responses to CMV and LPS were significantly lower in TB-IRIS patients compared to non-IRIS controls (p = 0.039 and p = 0.016, respectively). TB-IRIS patients also showed lower CMV responses compared to HIV-TB+ controls (p = 0.027), and lower LPS responses compared to HIV-TB+ and HIV-TB- controls (p<0.001 and p = 0.001, respectively). In contrast to TB-IRIS patients, non-IRIS controls showed a significantly recovered response to LPS compared to pre-ART (p = 0.015), which was still lower compared to HIV-TB+ controls (p = 0.034). Responses to influenza were not significantly different between TB-IRIS patients and non-IRIS controls. Responses to influenza did not recover within the first weeks of ART for either TB-IRIS patients or for non-IRIS controls compared to those in HIV-TB+ (p≤0.003) and HIV-TB- controls (p≤0.015) at every time point.

### Innate cytokine production upon stimulation with CMV, PPD and LPS

**Figure 2:**
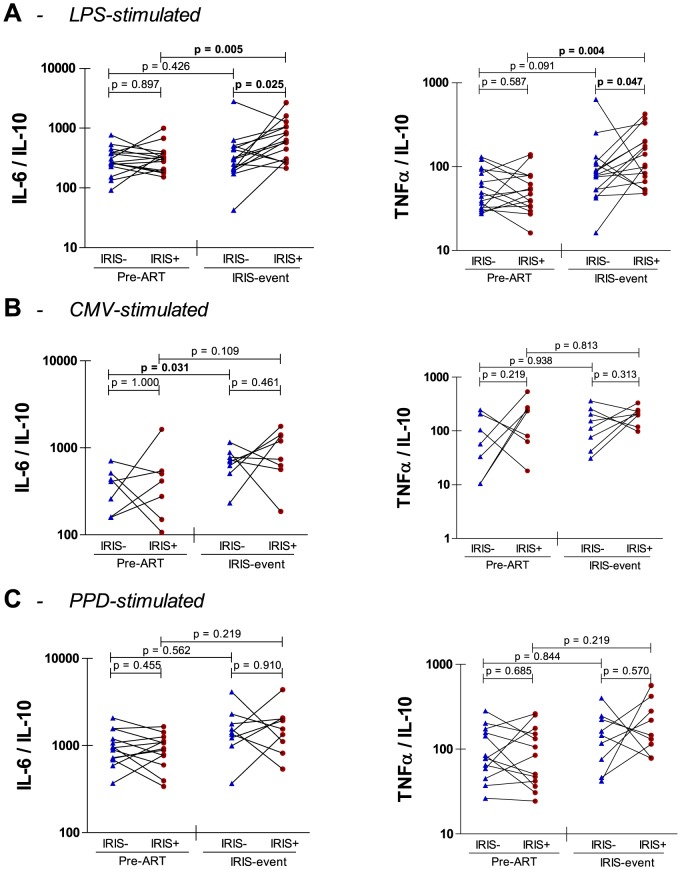
Pro- to anti-inflammatory ratios of innate cytokine production in TB-IRIS. Dots on these graphs represent cytokine ratios in PBMC supernatants after stimulation with LPS (A), CMV (B) and PPD (C). Dots connected with full lines represent matched pairs of TB-IRIS patients (IRIS+) with non-IRIS controls (IRIS-). Horizontal capped lines represent statistical comparisons between matched patients or between time points. A, pre-ART n = 16, IRIS event n = 16; B, pre-ART n = 7, IRIS event n = 8; C, pre-ART n = 13, IRIS event n = 9. A Wilcoxon signed-rank test was used to calculate p values between HIV patients. The level of significance was set to p<0.05.

**Figure 3:**
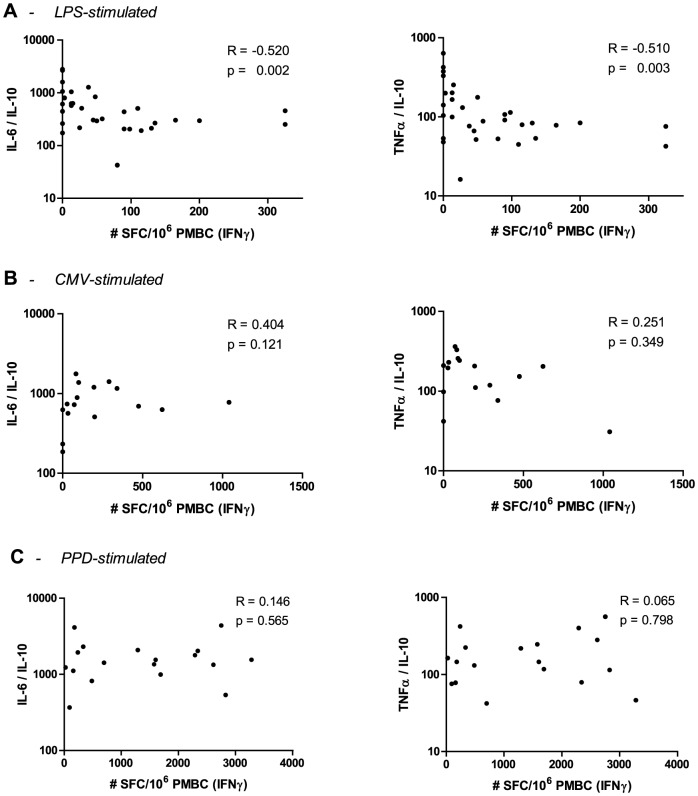
Correlation of cytokine ratios to IFNγ responses. Graphs represent the correlation between IFNγ responses and IL-6/IL-10 or TNFα/IL-10 ratios after stimulation with LPS (A), CMV (B) or PPD (C). Dots represent both TB-IRIS patients and non-IRIS controls during TB-IRIS or corresponding control time point. The level of significance was set to p<0.05.

We next explored whether production of innate cytokines could provide an explanation for the delayed reconstitution of CMV and LPS responses in TB-IRIS patients. To this end, we analysed levels of the pro-inflammatory cytokines IL-12, TNFα and IL-6 and of the anti-inflammatory cytokine IL-10 in ELISpot supernatants after stimulation with CMV, PPD and LPS, but found no differences between TB-IRIS patients and non-IRIS controls (Table 2). Systemic inflammation can be marked by increases in pro-inflammatory cytokines as well as by decreases in anti-inflammatory cytokines and their net balance has been shown to determine the clinical outcome of inflammation [33]–[37]. Accordingly, we explored IL-6/IL-10 and TNFα/IL-10 ratios in TB-IRIS patients and non-IRIS controls (Figure 2). After LPS stimulation, we observed significantly higher IL-6/IL-10 (p = 0.025) and TNFα/IL-10 (p = 0.047) ratios in TB-IRIS patients compared to non-IRIS controls (Figure 2A). These differences corresponded to significant increases of IL-6/IL-10 (p = 0.005) and TNFα/IL-10 (p = 0.004) ratios from pre-ART to IRIS event in TB-IRIS patients but not in non-IRIS controls. Interestingly, IL-6/IL-10 and TNFα/IL-10 ratios correlated inversely with IFNγ ELISpot responses to LPS in the total HIV+/TB+ patient population (Figure 3). Stimulation with CMV or PPD did not yield significant differences in cytokine ratios between patient groups (Figure 2B&C) nor resulted in significant correlations between IFNγ ELISpot responses and cytokine ratios (Figure 3B&C).

**Table 2:** In vitro cytokine production in response to CMV, PPD or LPS stimulation in TB-IRIS patients and non-IRIS controls.

	Pre-ART			IRIS event			Change over time (p^a^)	
	TB-IRIS	Control	p^a^	TB-IRIS	Control	p^a^	TB-IRIS	Control
**CMV stimulation** b								
IL-12p70 (pg/ml)	1.3 (0.6–2.4)	0.9 (0.6–2.4)	0.866	2.6 (1.1–3.1)	2.2 (1.1–3.1)	0.725	0.225	0.107
IL-6 (pg/ml)	207.9 (64.1–1633.8)	103.3 (32.2–523.6)	0.735	1491.5 (118.9–2538.4)	933.7 (295.1–3126.7)	0.311	0.237	0.028
TNFα (pg/ml)	135.4 (37.9–338.8)	49.2 (2.1–145.3)	0.753	279.5 (52.4–354.0)	159.8 (100.65–380.05)	0.462	0.091	0.091
IL-10 (pg/ml)	0.6 (0.5–1.0)	0.2 (0.2–1.2)	0.237	1.4 (0.4–1.9)	1.6 (0.35–4.15)	1.000	0.753	0.043
**PPD stimulation** c								
IL-6 (ng/ml)	16.3 (9.0–35.9)	15.9 (8.9–19.1)	0.345	54.2 (11.1–71.1)	33.1 (27.7–40.6)	0.594	0.128	0.116
TNFα (ng/ml)	1.3 (0.7–2.0)	1.0 (0.6–2.5)	0.221	3.8 (1.2–9.1)	3.4 (1.0–6.2)	0.953	0.043	0.463
IL-10 (pg/ml)	16.8 (10.5–47.0)	17.3 (8.5–30.8)	0.152	16.3 (7.8–47.5)	23.5 (8.8–30.1)	0.859	0.735	0.463
**LPS stimulation** d								
IL-12p70 (pg/ml)	4.8 (2.3-7.6)	3.9 (3.0–5.9)	0.453	4.7 (3.1–9.5)	5.4 (3.0–7.5)	0.277	0.315	0.900
IL-6 (ng/ml)	25.7 (10.9–58.6)	22.2 (10.2–36.6)	0.215	22.9 (12.2–106.8)	25.6 (13.7–42.6)	0.352	0.156	0.245
TNFα (ng/ml)	4.9 (1.8–9.3)	4.2 (2.0–7.6)	0.352	5.0 (2.0–25)	7.5 (3.0–13.4)	0.469	0.307	0.363
IL-10 (pg/ml)	72.6 (31.4–304.6)	60.0 (23.2–215.2)	0.408	47.1 (18.9–121.6)	97.8 (41.2–207.7)	0.215	0.334	0.683

Values are shown as median values with interquartile range. The level of significance was set to p<0.05 for all tests.

*^a^* Wilcoxon signed-rank test. Due to limited availability of PBMCs, the number of patients differed;

*^b^* 7 at pre-ART and 8 during IRIS event,

*^c^* 13 at pre-ART and 9 during IRIS event,

*^d^* 16 at pre-ART and 16 during IRIS event.

## Discussion

TB-IRIS could involve an amplified immune response to TB bacilli or their residual antigens [11], [13]. Here, we aimed to study the recovery of antigen-specific responses of HIV-TB patients who developed TB-IRIS during ART. To that end, we compared IFNγ responses of PBMCs to a panel of TB-associated antigens and recall antigens between TB-IRIS patients and non-IRIS controls, matched for CD4 count, age and sex. In addition, we explored innate cytokine responses to CMV, PPD and LPS. We report a disturbed reconstitution of the IFNγ response to CMV and LPS during TB-IRIS, without an excessive IFNγ response to TB-antigens. In addition, we observed a pro-inflammatory shift in the innate cytokine balance upon LPS stimulation during TB-IRIS.

TB-IRIS patients showed lower IFNγ responses to the recall antigen CMV and to LPS during TB-IRIS, compared to non-IRIS controls. Unlike CMV and LPS, however, we did not observe significantly increased or decreased IFNγ responses to any of the TB-antigens, nor to Influenza in TB-IRIS patients. These results are in line with a number of previous studies [14], [19]–[21]. However, our findings are not in agreement with previous reports of elevated PPD-responses during TB-IRIS [14]–[18]. The magnitude of IFNγ ELISPOT responses to TB-antigens has been associated to the antigen-load, duration of TB-treatment and the extent of immune-suppression in HIV patients [38]–[40]. All three of these factors have been identified as risk-factors for TB-IRIS which could potentially influence immunological measurements, consequently leading to discrepancies across studies. In the present study, we minimize this potential bias by directly comparing (time-)matched patients under very similar clinical conditions, possibly explaining the discrepancy with studies that observed elevations in TB-associated responses. Together, our results suggest that the conditions associated with TB-IRIS, such as a high TB-antigen load and persistent inflammation [41]–[44], could disturb the reconstitution of recall responses (to CMV in this case), rather than cause an excessive TB-specific IFNγ response. In addition, the lowered response to LPS could point towards a role of the innate immune system.

Since we previously reported a significant rise in IL-6 plasma levels from pre-ART to TB-IRIS event, which lead to higher IL-6 levels compared to non-IRIS controls [26], we next hypothesised that TB-IRIS might result from an aberrant innate immune response. In contrast to our previous in vivo measurements, we found no significant differences in the in vitro production of pro- and anti-inflammatory cytokines between matched TB-IRIS patients and non-IRIS controls after exposure to CMV, PPD and LPS. In addition to absolute levels, however, the balance between pro-inflammatory and anti-inflammatory cytokine levels has been shown to drive systemic inflammation [33]–[37]. Accordingly, we found that TB-IRIS was associated with a pro-inflammatory shift in the IL-6/IL-10 and TNFα/IL-10 ratios after stimulation with LPS, but not CMV or PPD. An increase in the IL-6/IL-10 ratio, caused by a decrease in IL-10, has previously been associated to the severity of systemic inflammatory response syndrome in patients with sepsis [34]. Of note, the IL-10 levels upon LPS stimulation in the current study were also somewhat lower during TB-IRIS. Although this difference did not reach statistical significance, it could have shifted the cytokine balance towards the pro-inflammatory side. In line with our findings, TB-IRIS patients from our cohort have previously been shown to have a pro-inflammatory monocyte-gene expression profile that is also perturbed in pattern recognition receptor pathways [45]. Another study previously reported elevated TNFα production during IRIS upon TLR2 stimulation with lipomannan, without an equivalent rise in IL-10 [16]. In the present study, we report a similar cytokine imbalance in the TLR4 branch of innate cytokine production. One could therefore argue that a disturbed equilibrium between pro- and anti-inflammatory cytokine-production upon TLR stimulation is implicated in the high degree of inflammation seen in TB-IRIS. This preferential involvement of TLRs in TB-IRIS could also explain why no cytokine shifts were observed after CMV- or PPD-stimulation, since these antigens preferentially induce an adaptive response via the major histocompatibility complex class II/T cell receptor pathway.

Intriguingly, the unbalanced cytokine ratios were inversely correlated to the LPS-induced IFNγ responses. This finding is somewhat contradictory, given the fact that IL-6, TNFα and IFNγ are all pro-inflammatory cytokines. However, IL-6 and TNFα are directly produced by monocytes after LPS stimulation, while IFNγ is not. Rather, LPS-induced IFNγ originates from T cells and NK cells in response to monocyte derived cytokines [46], [47]. We hypothesise that the cytokine ratio shifts result from aberrant monocyte behaviour in these patients, given the association of monocyte dysfunction with chronic HIV infection [48], [49]. In fact, aberrant monocyte behaviour has previously been suggested to play a role in TB-IRIS [50] and is in line with our hypothesis on the role of TLRs in TB-IRIS. Since the balance between monocyte-derived cytokines seems to be disturbed upon TLR stimulation, we speculate that this negatively affected the subsequent induction of IFNγ.

One limitation of our study was that patients and controls were not tested for CMV and influenza infection status, potentially complicating the interpretation of the CMV and influenza ELISPOT responses that we measured. This may not have been a major problem for CMV, which reaches a high seroprevalence in sub Saharan Africa [51], [52]. While influenza is clearly present in sub Saharan Africa [53], [54], limited availability of epidemiological data make it difficult to assess the expected recall response of our patients to this antigen.

Taken together, our data provide no evidence of an excessive IFNγ response to TB-associated antigens or other common recall antigens during TB-IRIS. In fact, TB-IRIS was associated with a disturbed reconstitution of the IFNγ responses to CMV and LPS. For LPS, this was linked with a pro-inflammatory shift in the innate cytokine balance. Together, our data provide evidence for a prominent role for innate immune inflammation and possibly monocyte dysfunction in TB-IRIS pathogenesis. Resolving the immune responses leading to TB-IRIS pathogenesis could provide novel targets for treatment and prevention.
